# The Effect of Glutamate Receptor Agonists on Mouse Retinal Astrocyte [Ca^2+^]_i_

**DOI:** 10.1155/2016/8178162

**Published:** 2016-06-19

**Authors:** Stephanie N. Blandford, William H. Baldridge

**Affiliations:** ^1^Retina and Optic Nerve Research Laboratory, Dalhousie University, Halifax, Nova Scotia, Canada B3H 4R2; ^2^Department of Medical Neuroscience, Dalhousie University, Halifax, Nova Scotia, Canada B3H 4R2; ^3^Department of Ophthalmology & Visual Sciences, Dalhousie University, Halifax, Nova Scotia, Canada B3H 4R2

## Abstract

Calcium-imaging techniques were used to determine if mouse retinal astrocytes* in situ* respond to agonists of ionotropic (*α*-amino-3-hydroxy-5-methyl-4-isoxazolepropionic acid, AMPA; N-methyl-D-aspartate, NMDA) and metabotropic (*S*-3,5-dihydroxyphenylglycine, DHPG;* trans*-1-amino-1,3-cyclopentanedicarboxylic acid, ACPD) glutamate receptors. In most cases we found no evidence that retinal astrocyte intracellular calcium ion concentration ([Ca^2+^]_i_) increased in response to these glutamate agonists. The one exception was AMPA that increased [Ca^2+^]_i_ in some, but not all, mouse retinal astrocytes* in situ*. However, AMPA did not increase [Ca^2+^]_i_ in mouse retinal astrocytes* in vitro*, suggesting that the effect of AMPA* in situ* may be indirect.

## 1. Introduction

Historically astrocytes were regarded as structural components of the nervous system parenchyma, with few functional attributes. This view changed when astrocytes* in vitro* and* in situ* were found to possess receptors for many neurotransmitters including glutamate, adenosine, *γ*-aminobutyric acid (GABA), and epinephrine (for review see [1–3]). Activation of astrocyte neurotransmitter receptors commonly results in an increase in cytosolic calcium concentration ([Ca^2+^]_i_) that could lead to calcium-dependent release of chemical transmitters, termed gliotransmitters (for review see [4]).

The effect of glutamate, the predominant excitatory neurotransmitter in the CNS, on astrocytes has been well documented in the brain. Early experiments using acutely isolated hippocampal astrocyte cultures showed that application of glutamate resulted in an initial spike-like rise in [Ca^2+^]_i_ followed by a sustained [Ca^2+^]_i_ increase [5]. Further, astrocytes in hippocampal slices showed increased [Ca^2+^]_i_ following exposure to glutamate released from Shaffer collaterals upon stimulation [6, 7]. Astrocyte activation and calcium mobilization caused by glutamate lead to the subsequent release of gliotransmitters, such as adenosine triphosphate (ATP) and D-serine [8, 9]. Numerous studies have revealed that both metabotropic and ionotropic glutamate receptors are expressed by brain astrocytes and that activation of these receptors commonly results in measureable increases in [Ca^2+^]_i_ [1, 7, 9–12].

There are two types of macroglia in the mammalian retina: astrocytes and Müller cells. Anatomically, astrocytes are confined to the nerve fibre layer (NFL), whereas Müller cells run radially from the vitreal surface, or the inner limiting membrane, to the outer limiting membrane. Several studies have suggested that Müller cells are involved in neurovascular coupling mediated by ATP, both as the recipient of the neuron-to-glia signal and as the source of ATP released from glia onto retinal vessels [13–16].

Glutamate is the primary excitatory neurotransmitter in the retina and, therefore, could potentially contribute to neuronal-glia signalling in the retina. Müller cells in some [17, 18] but not all [19] types of vertebrate retinas show increased [Ca^2+^]_i_ in response to glutamate or glutamate receptor agonists. It is not clear if retinal astrocytes respond to glutamate and if such responses involve an increase in [Ca^2+^]_i_. A study of rabbit retinal astrocytes* in situ* [20] demonstrated that glutamate and *α*-amino-3-hydroxy-5-methyl-4-isoxazolepropionic acid (AMPA) agonists evoked inward currents. Glutamate-evoked currents were blocked by the AMPA/kainate ionotropic glutamate receptor antagonist 6-cyano-7-nitroquinoxaline-2,3-dione (CNQX). N-Methyl-D-aspartate (NMDA) did not evoke currents in these astrocytes [20]. In contrast, when applied to rat retinal astrocytes* in situ*, glutamate did not evoke [Ca^2+^]_i_ increases [19].

In the present study we used calcium-imaging techniques to determine if mouse retinal astrocytes* in situ* respond to agonists of ionotropic and metabotropic glutamate receptors. In most cases we find no evidence to suggest that retinal astrocytes are responsive to glutamate agonists. The one exception was AMPA that increased [Ca^2+^]_i_ in some, but not all, mouse retinal astrocytes* in situ*. However, AMPA did not increase [Ca^2+^]_i_ in mouse retinal astrocytes* in vitro*, suggesting that the action of AMPA* in situ* may be indirect.

## 2. Materials and Methods

The Dalhousie University Committee on Laboratory Animals (UCLA) approved all protocols, and procedures were performed in accord with regulations established by the Canadian Council on Animal Care (CCAC). All chemicals were obtained from Sigma-Aldrich (Oakville, ON, Canada) unless stated otherwise.

### 2.1. Animals and Husbandry

C57BL/6 mice were purchased from Charles River (St. Constant, Quebec, Canada) and housed at the Carleton Animal Care Facility in the Tupper Medical building at Dalhousie University. Animals were kept in cages with up to four other mice and had access to food and water* ad libitum*.

### 2.2. Tissue Preparation

Animals were sacrificed by a lethal intraperitoneal injection of sodium pentobarbital (CDMV, Dartmouth, NS, Canada). Upon confirmation of death, the animal's eyes were removed quickly and placed in room temperature Hanks' Balanced Salt Solution (HBSS) bubbled with 100% oxygen and proton buffered with 10 mM HEPES (pH 7.4). Astrocytes* in situ* were loaded with calcium dye by electroporation. Calcium indicator dye (750 nL of a 20 mM fura-2 pentapotassium salt solution; Invitrogen, Burlington, ON, Canada) was injected into the eye through the optic disk using a Hamilton syringe. Tweezertrodes (BTX, Holliston, MA, USA) were positioned on the eye with the anode on the cornea and square wave current pulses were applied using the ECM 830 electroporation system (BTX). The eyes were electroporated with five square 20 ms current pulses of 30 V at 1 Hz. Retinas were isolated by careful dissection and the vitreous humour removed by gentle tweezing with forceps. Retinas were flat mounted on black filter paper (Millipore) with the vitreal surface facing up, secured in place with a platinum ring, and maintained for 10–15 minutes in oxygenated HBSS prior to being transferred to a microscope-mounted superfusion chamber for calcium imaging.

### 2.3. Preparation of Isolated Retinal Astrocyte Cultures

Isolated retinas from 2-3 C57BL/6 mice (Charles River) were added to 5 mL Mg^2+^- and Ca^2+^-free Dulbecco's Phosphate Buffered Saline (DPBS; Invitrogen) containing 56 *μ*L papain (16.5 units/mL, 29.4 units/mg of protein; Worthington) and 50 *μ*L 0.004% DNase (Worthington). Retinas were incubated in this solution at 37°C for 30 min. Following incubation, the papain solution was aspirated from the retinas. Next, 400 *μ*L of media composed of 45 mL Dulbecco's Modified Eagle Medium (DMEM; Invitrogen), 5 mL Fetal Bovine Serum (FBS; Hemoglobin ≤ 20 mg/dL), and 100 *μ*L Penicillin Streptomycin (Pen/Strep, 10,000 units/mL; Invitrogen), hereafter simply referred to as media, was added, and retinas were gently triturated. Glass coverslips (12 mm) were washed overnight in 70% ethanol on a shaker. The next morning, they were rinsed three times in distilled water and autoclaved for 20 min. Autoclaved coverslips were placed in Nunc 4-well plates and coated with Poly-D-Lysine (PDL; 10 *μ*g/*μ*L) for one hr and then rinsed three times in distilled water. Cells were plated on the prepared coverslips and stored in wells containing media in an incubator at 37°C for at least 14 days to allow cells to grow. Media were changed every 2-3 days. For calcium imaging individual coverslips were incubated in 5 *μ*M fura-2-acetoxymethyl ester (AM; Invitrogen) dissolved in 100% oxygenated HBSS (10 mM HEPES, pH 7.4, 1 *μ*g/mL pluronic acid) for one hour before being transferred to a superfusion chamber mounted on a microscope.

### 2.4. Calcium Imaging

Single retinas or coverslips containing cultured retinal astrocytes were placed in a superfusion chamber (~750 *μ*L volume) and superfused with 100% oxygenated HBSS (10 mM HEPES, pH 7.4) at a rate of ~2 mL/min. Retinas were imaged with a charge-coupled device (CCD) camera (Sensicam, PCO, Germany) connected to a Zeiss Axioskop microscope equipped with a 40x water immersion objective and recorded using Axon Imaging Workbench 4 software (Molecular Devices, Sunnyvale, CA, USA). Images were typically acquired at a frequency of one frame per 20 sec, increased to one frame per 5 sec during periods of drug applications. Ratiometric fura-2 dyes (*K*_*d*_ = 224 nM) were used, and image pairs at 340 and 380 nm excitation (510 nm emission) were collected.

### 2.5. Experimental Protocol

In whole mount retinas, astrocytes loaded with fura-2 were identified based on stereotypical morphological characteristics [19], including a flattened cell body with a series of radiating processes, forming a layer, positioned within the nerve fibre layer, that can be identified in whole mount preparations as just superficial (vitreal) to neurons (also loaded somewhat with fura-2) in the ganglion cell layer, and (frequently, but not always) forming perivascular endfeet on retinal blood vessels. The effect of ionotropic glutamate receptor agonist on astrocyte calcium levels was tested by exposing astrocytes to ionotropic glutamate receptor agonists (10 *μ*M AMPA or 50 *μ*M NMDA), the general metabotropic glutamate receptor agonist* trans*-1-amino-1,3-cyclopentanedicarboxylic acid monohydrate (ACPD, at 50 *μ*M), or the group I metabotropic agonist (*S*)-3,5-dihydroxyphenylglycine (DHPG, at 50 *μ*M) dissolved in the superfusate solution, for 30 sec. Each preparation was treated with one agonist two to four times. To assess cell viability, and to contrast responses to the effect of the glutamate agonists, the calcium response induced by 10 *μ*M ATP was also tested in most astrocytes studied.

### 2.6. Data Analysis

Using Axon Imaging Workbench (AIW), identified astrocytes were circled* post hoc *as a region of interest (ROI). The software provided measures of fluorescence ratio with respect to time for each ROI. Statistical analysis was conducted using Prism 6 (GraphPad, La Jolla, CA, USA).

Baseline ratios were analyzed to assess fluctuations (noise) over 3 min periods prior to agonist treatments. Agonist-induced calcium dynamics were determined as the peak change (Δ*f*) from baseline (*f*) during a 3 min period following the onset of a 30 s agonist application. Peak Δ*f* for each agonist application was averaged for each cell, and averages for each cell were used to calculate the mean peak Δ*f* of each retina or coverslip. For each condition mean baseline, agonist- and ATP-induced Δ*f* values were compared using the Kruskal-Wallis test and Dunn's multiple comparisons test.

## 3. Results

### 3.1. Loading Astrocytes with Calcium Indicator Dye

**Figure 1:**
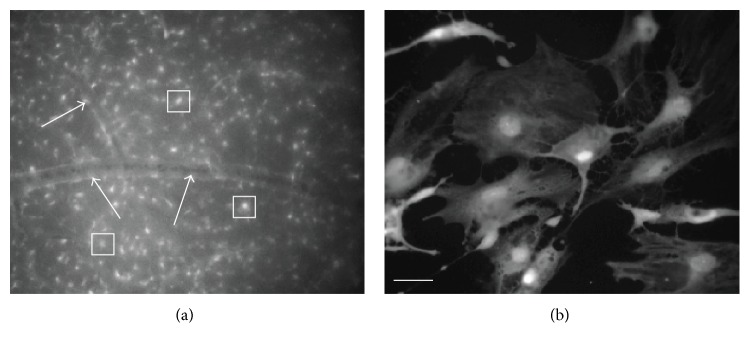
Photomicrographs illustrating loading of astrocytes with fura-2 and imaged at 380 nm excitation and 510 nm emission. (a) Astrocytes in the retinal whole mount preparation (*in situ*) loaded with fura-2 by electroporation. Numerous astrocytes are loaded; some examples are highlighted within boxes. A blood vessel can been seen with fura-2-loaded presumptive perivascular endfeet (arrows). (b) Astrocytes* in vitro* loaded with fura-2. Scale bar (for both (a) and (b)) 50 *μ*m.

Electroporation has proven an effective means to load retinal neurons* in situ* with calcium indicator dye [21–24]. We made the serendipitous discovery that by reversing the polarity of the electroporation electrodes on the eye (placing the anode on the anterior pole and the cathode on the posterior pole) there was strong loading of astrocytes with calcium indicator dye (Figure 1(a)). Astrocytes loaded with dye (in this case fura-2) were identified based on stereotypical morphological characteristics [19] including a flattened cell body with a series of radiating processes, forming a layer, positioned within the NFL, that can be identified in whole mount preparations near to blood vessels with perivascular endfeet contacts on retinal blood vessels. Isolated astrocytes were loaded with fura-2 by conventional means (incubation in solution containing membrane-permeable fura-2 AM; Figure 1(b)).

### 3.2. Effect of AMPA and NMDA on [Ca^2+^]_i_ in Retinal Astrocytes* In Situ*

**Figure 2:**
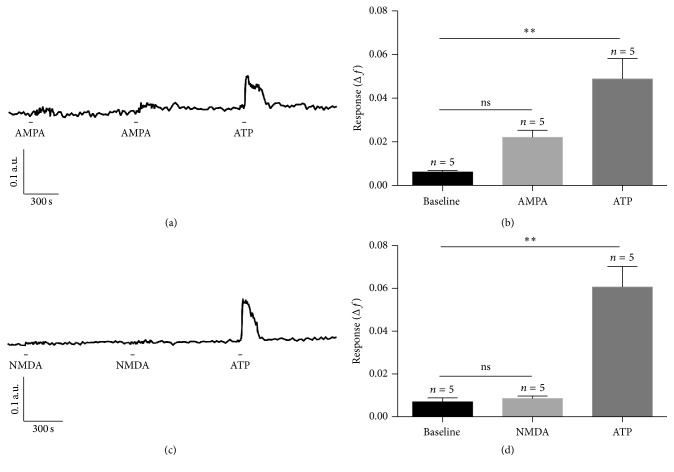
Effect of ionotropic glutamate receptor agonists AMPA and NMDA on retinal astrocytes [Ca^2+^]_i_ *in situ*. (a) Representative trace showing fura-2 fluorescence ratio of an astrocyte treated twice with 10 *μ*M AMPA followed by 10 *μ*M ATP. The astrocyte showed a small, but discernable, response to both treatments with 10 *μ*M AMPA and a larger response to 10 *μ*M ATP. (b) The mean response elicited by AMPA was not significantly different from baseline, but the mean response elicited by ATP was. (c) Representative trace showing fura-2 fluorescence ratio of an astrocyte treated twice with 10 *μ*M NMDA followed by 10 *μ*M ATP. The astrocyte responded to ATP, but not NMDA. (d) Mean response elicited by NMDA application was not significantly different from baseline, while mean response elicited by ATP was. Responses are reported as the average peak change (Δ*f*) from baseline (*f*) during a 3 min period following 30 s agonist applications. Error bars are SEM, *n* = number of retinas studied, ns: not significant; ^*∗∗*^ *p* < 0.01 at *α* = 0.05 as determined by Dunn's multiple comparisons test following Kruskal Wallis ANOVA.

Bath application of 10 *μ*M AMPA elicited an increase of fura-2 fluorescence ratio in many but not all mouse retinal astrocytes studied* in situ*.  Figure 2(a) shows a trace from a representative astrocyte treated twice with 10 *μ*M AMPA and, at the conclusion of the experiment, 10 *μ*M ATP. The response to ATP was robust whereas the response to AMPA was smaller, but discernable. Mean data from all astrocytes studied in 5 different retinas (139 astrocytes) are illustrated in Figure 2(b). Although the mean peak response to both AMPA and ATP was increased relative to baseline (a measure of the fluctuations over 3 min prior to agonist treatment), and the results of the Kruskal-Wallis test indicated that there was a significant difference in mean peak Δ*f* (baseline, AMPA- and ATP-treated; *H* = 11.58; *p* < 0.0001 at *α* = 0.05), Dunn's multiple comparison tests revealed that there was only a significant difference between baseline and the mean peak response to ATP (Figure 2(b)). Another way to assess this data [10, 25] is to consider how many astrocytes responded to treatment, defined by reaching a minimum increase of calcium dye fluorescence signal. We defined a responding cell as one that showed an increase in fura-2 fluorescence ratio ≥ 0.02 from mean baseline during treatment (and this exceeded 2 standard deviations associated with mean baseline fluorescence). 45% of all astrocytes studied showed an increase (Δ*f*) of fura-2 fluorescence ratio ≥ 0.02 in response to 10 *μ*M AMPA. All cells (100%) responded to 10 *μ*M ATP. The number of cells that responded (Δ*f* ≥ 0.02) to ATP but not AMPA (55% of all cells studied) limits the comparison of the mean data. That a significant number of cells (45% of all cells studied) responded to AMPA suggests that at least some astrocytes show increases in [Ca^2+^]_i_ in response to AMPA application* in situ*.

Bath applications of 50 *μ*M NMDA typically did not elicit a response from mouse retinal astrocytes* in situ* (Figures 2(c) and 2(d)).  Figure 2(c) shows a trace from a representative cell treated twice with 50 *μ*M NMDA and then 10 *μ*M ATP. The mean peak Δ*f* measured from the 5 retinas studied (179 astrocytes) during NMDA treatment was indistinguishable from baseline (Figure 2(d)). The Kruskal-Wallis test revealed that there was a significant difference between mean peak Δ*f* (baseline, NMDA-, and ATP-treated; *H* = 9.620, *p* = 0.0018), but this difference was due solely to the effect of ATP (Dunn's multiple comparisons test; Figure 2(d)). Of the 179 astrocytes studied, only 3 cells (2%) showed an increase in fura-2 fluorescence ratio of ≥0.02 during treatment with 50 *μ*M NMDA. All cells (100%) responded to 10 *μ*M ATP.

### 3.3. Effect of DHPG and ACPD on [Ca^2+^]_i_ in Retinal Astrocytes* In Situ*

**Figure 3:**
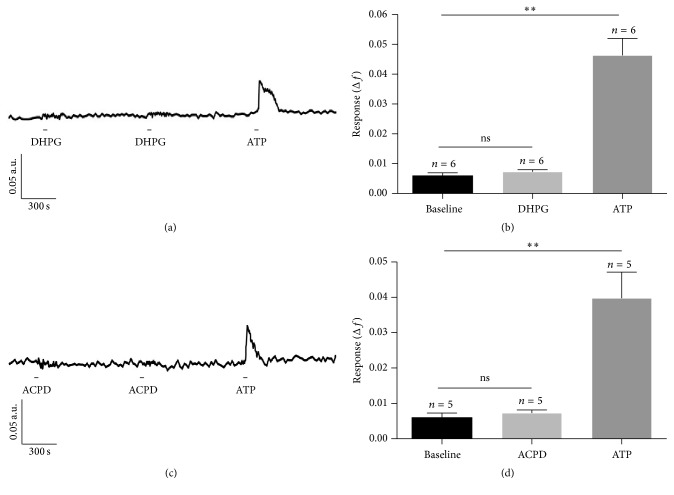
Effect of metabotropic glutamate receptor agonists DHPG and ACPD on retinal astrocytes [Ca^2+^]_i_ *in situ*. (a) Representative trace showing fura-2 fluorescence ratio of an astrocyte treated twice with 50 *μ*M DHPG followed by 10 *μ*M ATP. The astrocyte responded to ATP, but not DHPG. (b) The mean response elicited by DHPG was not significantly different from baseline, but the mean response elicited by ATP was. (c) Representative trace showing fura-2 fluorescence ratio of an astrocyte treated twice with 50 *μ*M ACPD followed by 10 *μ*M ATP. The astrocyte responded to ATP, but not ACPD. (d) Mean response elicited by ACPD application was not significantly different from baseline, while mean response elicited by ATP was. Error bars are SEM, *n* = number of retinas studied, ns: not significant; ^*∗∗*^ *p* < 0.01 at *α* = 0.05 as determined by Dunn's multiple comparison test following Kruskal-Wallis ANOVA.

Bath application of the group I metabotropic glutamate receptor agonist DHPG (50 *μ*M) did not elicit a significant response from mouse retinal astrocytes* in situ* (Figures 3(a) and 3(b)).  Figure 3(a) shows a trace from a representative astrocyte treated twice with 50 *μ*M DHPG followed by treatment with a single treatment with 10 *μ*M ATP. ATP increased the ratio of fura-2 fluorescence but DHPG had no effect. A similar result was found for mean data from astrocytes studied in 5 different retinas (364 cells) (Figure 3(b)). Statistical analysis (Figure 2(b)) indicated that there was a significant difference between baseline and the mean peak response to ATP (Kruskal-Wallis *H* = 12.12, *p* = 0.0023 followed by Dunn's multiple comparison tests). Of the 364 astrocytes studied, only 8 cells (2%) showed an increase in fura-2 fluorescence ratio of ≥0.02 during treatment with 50 *μ*M DHPG. All cells (100%) responded to 10 *μ*M ATP.

Similarly, bath applications of the general metabotropic glutamate receptor agonist ACPD (50 *μ*M) did not elicit a significant response from mouse retinal astrocytes* in situ* (Figures 3(c) and 3(d)). The Kruskal-Wallis test revealed that there was a significant difference between mean peak Δ*f* (baseline, ACPD-, and ATP-treated; *H* = 9.62, *p* = 0.0081), but this difference was due solely to the effect of ATP (Dunn's multiple comparisons test; Figure 2(d)). Of the 179 astrocytes studied, only 4 cells (2%) showed an increase in fura-2 fluorescence ratio of ≥0.02 during treatment with 50 *μ*M ACPD. All cells (100%) responded to 10 *μ*M ATP.

### 3.4. Effect of AMPA and NMDA on Retinal Astrocytes* In Vitro*

**Figure 4:**
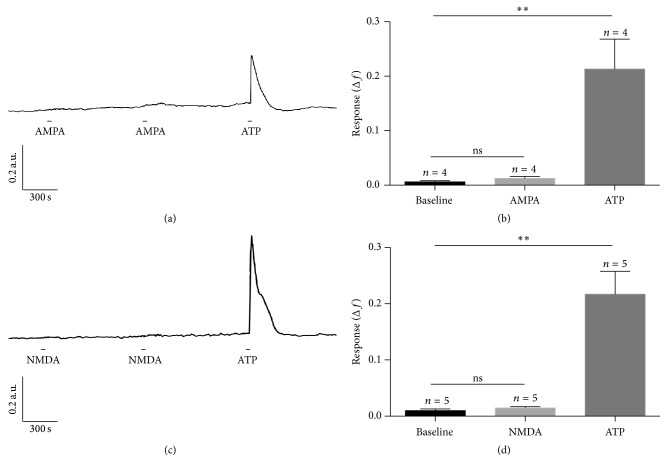
Effect of ionotropic glutamate receptor agonists AMPA and NMDA on retinal astrocytes [Ca^2+^]_i_ *in vitro*. (a) Representative trace showing fura-2 fluorescence ratio of an astrocyte treated twice with 10 *μ*M AMPA followed by 10 *μ*M ATP. The astrocyte responded to ATP, but not AMPA. (b) The mean response elicited by AMPA was not significantly different from baseline, but the mean response elicited by ATP was. (c) Representative trace showing fura-2 fluorescence ratio of an astrocyte treated twice with 10 *μ*M NMDA followed by 10 *μ*M ATP. The astrocyte responded to ATP, but not NMDA. (d) Mean response elicited by NMDA application was not significantly different from baseline, while mean response elicited by ATP was. Error bars are SEM, *n* = number of retinas studied, ns: not significant; ^*∗∗*^ *p* < 0.01 at *α* = 0.05 as determined by Dunn's multiple comparison test following Kruskal-Wallis ANOVA.

Thus far, the only glutamate receptor agonist that produced an increase in fura-2 fluorescence ratio* in situ* (in 45% of cells tested) was AMPA. To determine if the effect of AMPA* in situ* was due to a direct effect on astrocytes, we tested the effect of AMPA (and also NMDA) on astrocytes* in vitro*. Bath application of 10 *μ*M AMPA or 50 *μ*M NMDA did not elicit a significant response from isolated mouse retinal astrocytes (Figure 4). Figures 4(a) and 4(c) show traces from representative cells treated twice with AMPA or NMDA, respectively, and then with 10 *μ*M ATP. In both cases ATP increased the ratio of fura-2 fluorescence, but neither AMPA nor NMDA had an effect. Statistical analysis of the mean data revealed differences between baseline and the peak mean Δ*f* response to ATP (Figure 4(b), Kruskal-Wallis *H* = 8.769, *p* = 0.0012; Figure 4(d), *H* = 9.980; *p* = 0.0009). The lack of an effect of AMPA on astrocytes* in vitro* suggests that the action of AMPA* in situ* may not be direct.

## 4. Discussion 

The results of the present study provide evidence that mouse retinal astrocyte [Ca^2+^]_i_, assessed by fura-2 imaging techniques, is not increased by the ionotropic and metabotropic glutamate receptor agonists NMDA, DHPG, and ACPD. Mean peak fura-2 fluorescence ratios during agonist applications were not different than fluctuations observed under baseline conditions. The one exception was AMPA. Although the increase in peak mean fura-2 fluorescence ratio, compared to baseline, was not statistically significant, about half (45%) of all the cells studied showed an increase in fluorescence of ≥0.02. (The number of such responding cells in the case of each of the other agonists was only 2%.) We concluded, therefore, that AMPA increases [Ca^2+^]_i_ in response to AMPA application* in situ*. To determine if the effect of AMPA was due to a direct effect on astrocytes, we examined the effect of AMPA on astrocytes* in vitro*. AMPA did not elicit a significant response from isolated mouse retinal astrocytes suggesting that the effect of AMPA* in situ* could be indirect.

Previous research has shown that astrocytes from the brain not only express ionotropic glutamate receptors [1–3, 5–7, 9–12] but also respond to ionotropic glutamate receptor agonists, both* in vitro *and* in situ*, including AMPA and NMDA [10, 26, 27]. In contrast, there are no studies examining the expression of glutamate receptor types expressed by retinal astrocytes and only two studies that have examined the effect of glutamate or AMPA/kainate glutamate receptor agonists on retinal astrocytes. Clark and Mobbs [20] reported that glutamate and AMPA/kainate receptor agonists evoked inward currents in rabbit retinal astrocytes* in situ*. This same study showed that glutamate-induced inward currents in retinal astrocytes were blocked by the AMPA/kainate receptor antagonist CNQX. In contrast, Newman and Zahs [19] reported that glutamate had no effect on rat retinal astrocyte [Ca^2+^]_i_ studied* in situ*, although glutamate did potentiate increases of [Ca^2+^]_i_ produced by other stimuli. Our results are consistent with the results of Clark and Mobbs [20] in that we were able to demonstrate an effect of AMPA on mouse retinal astrocytes* in situ*. However, because AMPA was without effect on mouse retinal astrocytes* in vitro*, it is possible that the effect of AMPA* in situ* was indirect. AMPA acting at retinal neurons or Müller cells could result in transmitter, such as ATP or acetylcholine, release that, in turn, could increase astrocyte [Ca^2+^]_i_ [28–30].

We found that NMDA did not increase [Ca^2+^]_i_ in mouse retinal astrocytes* in situ* or* in vitro*. A common concern when studying NMDA receptors is extracellular Mg^2+^ block of the NMDA receptor [31]. Schipke et al. [27] showed that NMDA-induced currents in mouse neocortical astrocytes were enhanced in Mg^2+^-free conditions, reduced at 4 mM Mg^2+^, and abolished at 10 mM Mg^2+^. However, the same study showed that NMDA-induced currents were present, though of less magnitude, under standard recording conditions (1.8 mM Mg^2+^). In fact, several studies have shown that glial NMDA receptors are much less (or not) sensitive to Mg^2+^ block [11, 32]. In our experiments, external Mg^2+^ was 0.8 mM. Whether or not Mg^2+^ block is an issue in mouse retinal astrocyte NMDA-dependent current or [Ca^2+^]_i_ increases deserves further investigation. Furthermore, the NMDA-induced calcium dynamics reported by Schipke et al. [27] were restricted to distal processes of the astrocytes and were not observed in the cell bodies. The focus of the present study was imaging of astrocyte somata, making it possible that responses occurred in distal processes that were not captured by our imaging.

Various types of ionotropic and metabotropic glutamate receptors are present on astrocytes in many different brain regions and, when activated, are often capable of increasing [Ca^2+^]_i_ (for review see [3]). That retinal astrocytes are different adds to evidence that astrocytes in the CNS are heterogeneous [33, 34]. In fact, even though retinal astrocytes are immigrants from the optic nerve [35], astrocytes in the mouse optic nerve show increases in [Ca^2+^]_i_ in response to glutamate and a variety of ionotropic and metabotropic agonists [30]. Although there was a small direct effect of glutamate, much of the effect of glutamate on optic nerve astrocyte [Ca^2+^]_i_ was blocked by purinergic P2 receptor antagonists suggesting that the effect of glutamate was mediated largely by an indirect effect mediated by ATP [30]. Nonetheless, our data suggest that the calcium responses of retinal and optic nerve astrocytes to glutamate agonists are different, underlining the heterogeneity of astrocytes in different regions of the CNS. Perhaps this should not come as a surprise given that the astrocytes studied in the optic nerve [30] were likely to have been mostly type 1A or type 2 astrocytes whereas retinal astrocytes are type 1B [36, 37].

Although our data suggest that glutamate is unlikely to be an important direct activator of retinal astrocytes, at least with respect to processes that would lead to elevated [Ca^2+^]_i_, it was clear that ATP was a potent and consistent elevator of [Ca^2+^]_i_ in mouse retinal astrocytes. Although the functional significance of elevated [Ca^2+^]_i_ in astrocytes remains controversial [38], it is possible that ATP-mediated increases of [Ca^2+^]_i_ in mouse retinal astrocytes are associated with activity that regulates neuronal or vascular functions. Although glutamate may not influence retinal astrocytes, ATP is not necessarily the only endogenous agent capable of increasing [Ca^2+^]_i_. As one example, we have preliminary evidence that the potent vasoactive peptide, endothelin-1, increases [Ca^2+^]_i_ in rat retinal astrocytes* in situ* [39].
